# Heneicosapentaenoic acid, a metabolite resulting from the α‐oxidation of α‐Hydroxy‐Docosahexaenoic Acid, exerts neuroprotection against Alzheimer’s disease and reduces astrocytic mitochondrial activity

**DOI:** 10.1002/alz.092724

**Published:** 2025-01-09

**Authors:** Joan Cabot, Sebastià Parets, Marc Miralles, Laura Trujillo‐Estrada, Antonia Gutierrez, Paula Fernández‐García, Victoria Lladó, Pablo V. Escriba, Manuel Torres

**Affiliations:** ^1^ University of the Balearic Islands, Palma de Mallorca Spain; ^2^ Laminar Pharmaceuticals, Palma de Mallorca Spain; ^3^ University of Malaga/CIBERNED/IBIMA, Málaga Spain; ^4^ University of California, Irvine, Irvine, CA USA; ^5^ Centro de Investigación Biomédica en Red sobre Enfermedades Neurodegenerativas (CIBERNED), Madrid Spain

## Abstract

**Background:**

Reactive astrocytes and neuron death by excitotoxicity are observed in Alzheimer’s disease (AD). DHA‐H (2‐hydroxy‐docosahexaenoic acid; 2‐OH‐C22:6 n‐3) is a molecule under development that has demonstrated therapeutic efficacy in both cellular and 5xFAD mouse model of AD. DHA‐H is metabolized through α‐oxidation to yield HPA (Heneicosapentaenoic acid; C21:5 n‐3). This metabolic conversion is considered necessary for the neuroprotective effect of DHA‐H.

**Methods:**

Cognitive evaluation was assessed by Radial Arm Maze in 5xFAD mice after 4 months of chronic oral treatment at a daily dose of 20 mg/kg (DHA‐H or HPA). The transformation of DHA‐H to HPA was analyzed in HEK293 cells and in 5xFAD mice by GC‐FID or GC‐MS (Gas Chromatography‐Flame Ionization Detector or Mass Spectrometry). Neuroprotective effect of DHA‐H and HPA was tested in neuron cells differentiated from SH‐SY5Y neuroblastoma cells that were stimulated with NMDA (N‐Methyl‐D‐Aspartate)/Ca to induce excitotoxicity in the presence or absence of oxythiamine (α‐oxidation inhibitor). ATP level reduction was measured with the ATP luminescence assay system ATPlite (Perkin Elmer) while mitochondrial function was assessed by measuring the oxygen consumption rate (OCR) with the Seahorse Cell Mito Stress Test kit (Agilent Technologies).

**Results:**

Chronic oral administration of DHA‐H or HPA prevented cognitive decline in 5xFAD mice. DHA‐H is converted to HPA via α‐oxidation in cells and mice. HPA accumulates in 5xFAD mice brain instead of DHA‐H after DHA‐H treatment. Both DHA‐H and HPA prevented NMDA/Ca‐induced neuron death and the neuroprotective effect of DHA‐H was partially reversed in the presence of oxythiamine. HPA decreased ATP production and mitochondrial respiration capacity in astrocytoma cells.

**Conclusions:**

The metabolic intermediate HPA, stemming from the α‐oxidation of DHA‐H, emerges as a prospective candidate for Alzheimer’s therapy. Although the mechanism of action behind its neuroprotective effect is still unknown, here we study the effect of HPA on ATP production and mitochondrial respiratory capacity in astrocytes as a possible mechanism.

